# lncRNAs AC156455.1 and AC104532.2 as Biomarkers for Diagnosis and Prognosis in Colorectal Cancer

**DOI:** 10.1155/2022/4872001

**Published:** 2022-10-13

**Authors:** Fei Kuang, De Luo, Mengjia Zhou, Juan Du, Jie Yang

**Affiliations:** ^1^Department of General Surgery, Changhai Hospital of the Second Military Medical University, Shanghai 200433, China; ^2^Department of Hepatobiliary Surgery, Affiliated Hospital of Southwest Medical University, Luzhou, Sichuan 646000, China; ^3^Department of Ultrasound, Seventh People's Hospital of Shanghai University of Traditional Chinese Medicine, Shanghai 200137, China; ^4^Department of Radiation Oncology, Peking University Third Hospital, Beijing 100191, China

## Abstract

**Background:**

There have been countless studies to date assessing specific oncogenic pathways in a range of tumor classes, but the role of N6-methyladenosine- (m6A-) related long noncoding RNAs (lncRNAs) in colorectal cancer (CRC) remains to be defined.

**Methods:**

We analyzed such m6A-related lncRNAs by conducting analyses of the Pearson correlation with information originating from the databank of The Cancer Genome Atlas (TCGA). The prognostic relevance of these lncRNAs in CRC was then assessed through a series of univariate Cox regression analyses, leading to the identification of two different m6A modification patterns; they are associated with clinical outcomes and have been used to estimate tumor immune microenvironment (TIME) by the CIBERSORT and ESTIMATE algorithms. We tested the expression of m6A-related lncRNAs in twelve pairs of colorectal cancer tissues and adjacent normal tissues from patients by qRT-PCR.

**Results:**

We discovered the prognostic risk signature composed of six m6A-related lncRNAs based upon TCGA data. When the overall survival of cases in the dataset of TCGA was investigated, the low-risk cases survived longer than the high-risk CRC cases in both the training and testing cohorts. ROC curves further indicated that m6A-related lncRNA prognostic signature (m6A-LPS) can effectively estimate the survival outcomes of patients in both of these cohorts. We found that lncRNAs AC156455.1 and AC104532.2 were upregulated in twelve colorectal cancer tissues compared with adjacent normal tissues using qRT-PCR.

**Conclusions:**

This data highlights that the lncRNAs AC156455.1 and AC104532.2 in CRC can be used as biomarkers for diagnostics and prognosis in CRC, demonstrating their potential as targets when designing novel immunotherapeutic regimens.

## 1. Introduction

Colorectal cancer (CRC) is well known as one of the most widespread and deadly cancers all around the globe, with roughly 400,000 and 212,000 new diagnoses and deaths annually, respectively [1]. Novel immunotherapy-based regimens developed in recent years can engage or enhance natural immunological pathways and cells within a treated host to aid in tumor cell clearance, and many of these immunotherapies have been effective when applied in combination with other treatments in individuals with a range of tumor classes [2–4]. However, immunotherapy is commonly beneficial to a poorly defined subset of cases, and therefore, novel strategies must be devised to determine which CRC patients are likely to respond to such treatment [2–5].

The most commonly found epigenetic modification on mRNAs and noncoding RNAs (ncRNAs) is N6-methyladenosine (m6A), which can alter the stability, translation, and splicing of modified RNAs in biologically important contexts [6, 7]. As a reversible and dynamic process, the modification of m6A is controlled through three primary classes of m6A-regulating proteins: “writers” (methyltransferases), “readers” (signal transducers), and “erasers” (demethylases) [8]. According to the latest studies, m6A modification has been identified as a mechanism capable of modulating oncogenesis in a range of tumor types. For example, METTL14 can drive cancer advancement and maintenance in acute myeloid leukemia by enhancing the self-renewal of leukemia stem cells [9], while knocking down FTO can compromise lung squamous cell carcinoma cell proliferative and invasive activity and the self-renewal of glioblastoma stem cells [10, 11]. Furthermore, YTHDF2 can decrease the EGFR mRNA stability in cancer cells of the liver, thereby compromising their proliferation [12].

Recent research has underscored the relevance of ncRNAs in the context of CRC both through the therapeutic delivery of small interfering RNAs (siRNAs) as well as through in-depth analyses of the functional importance of long ncRNAs (lncRNAs) [13–18]. How m6A-related lncRNAs function in the regulation of CRC onset and progression, however, has yet to be defined, and there have been few studies exploring the impact of m6A modification on lncRNA-mediated CRC development. By studying the association between m6A modifications and lncRNAs in this tumorigenesis-related setting, it may be possible to define novel biomarkers that can guide therapeutic targeting efforts.

Herein, we leveraged the dataset of The Cancer Genome Atlas (TCGA) and conducted a series of bioinformatics analyses to clarify the prognostic relevance of m6A-related lncRNAs in CRC cases. The overall aim of the current investigation is to conduct a systematic assessment of the relationship among m6A-related lncRNAs and CRC patient prognosis, the composition of tumor immune microenvironment (TIME), and expression of programmed death ligand 1 (PD-L1). Through clustering analyses and risk modeling, we were able to initiate an m6A-related lncRNA prognostic signature (m6A-LPS). Associations among clustering subgroups, PD-L1 status, immune scores, and immune cell infiltration were analyzed in light of m6A-LPS as a means of further understanding the association between this risk signature. We analyzed and confirmed m6A-related lncRNAs AC156455.1 and AC104532.2 as biomarkers for diagnostics and prognosis in CRC, which will provide a new foundation for future efforts to develop effective immunotherapeutic treatments for CRC.

## 2. Materials and Methods

### 2.1. Data Selection

Transcriptomic and clinical outcomes from cases with CRC were acquired from the database of TCGA (https://portal.gdc.cancer.gov/). Initially, 23 regulators of m6A RNA methylation were selected based upon prior reports [19, 20], including 8 writers (RBM15, VIRMA, METTL3, WTAP, METTL14, RBM15B, METTL16, and ZC3H13), 2 erasers (FTO and ALKBH5), and 13 readers (IGFBP2, YTHDC1-2, LRPPRC, YTHDF1-3, FMR1, HNRNPA2B1, IGF2BP1, IGFBP3, HNRNPC, and RBMX).

### 2.2. lncRNA Annotation

The GRCh38 lncRNA annotation file was downloaded from GENCODE to aid in TCGA data annotation. In total, 14,086 lncRNAs were identified in this dataset based upon Ensemble IDs.

### 2.3. Bioinformatics Analysis

Initially, m6A-related lncRNAs were identified in each dataset via a series of Pearson's correlation analyses (|Pearson′s R| > 0.5 and *p* < 0.001). Prognostic m6A-related lncRNAs were subsequently identified through univariate Cox regression analyses, with 101 shared m6A-related prognostic lncRNAs being identified through comparison of overlap among TCGA datasets.

CRC patients were classified into two cohorts via a k-means clustering approach based upon the expression of 23 m6A-modulating genes using the R “kmeans function” using the ConsensusClusterPlus package, with 1,000 computational permutations being performed to guarantee stability and reliability [21].

The ESTIMATE algorithm was implemented to compute immune scores with the “estimate” R package [22]. The CIBERSORT algorithm was further employed to approximate the levels of intratumor infiltration via 22 various immune cell populations according to the expression data of RNA (https://cibersort.stanford.edu/), with 1,000 permutations of this analysis being performed and samples with a CIBERSORT *p* < 0.05 being retained for comparisons of differential immune cell infiltration among CRC patient subgroups defined according to risk scores and clustering subtypes.

The “h.all.V6.2.symbols.gmt” hallmark gene set from MSigDB was employed for a GSEA approach conducted using the JACA program to compare differences among CRC patient subtypes with respect to survival outcomes. For this analysis, 1,000 random sampling permutations were employed. Gene set enrichment was described according to the false discovery rate (FDR) < 0.05 and NES.

An analysis of LASSO regression was performed within the TCGA training cohort to define m6A-related lncRNA-based prognostic risk signatures [23], with the most appropriate signature being selected via choosing the optimal penalty criterion (l) associated with the minimum 10-fold cross-validation. LASSO regression algorithm-derived coefficients were employed to develop a risk score model with the following general equation: *risk* *score* = *sum* of *coefficients* × *expression* *level* of *m*6*A* *regulator*. Risk scores were individually computed for each case in the training and testing cohorts, and cases were stratified into low- and high-risk groups, with median risk score values serving as the cutoff for patient stratification. Comparisons of patient outcomes were then made through Kaplan-Meier survival curves, while the sensitivity and specificity of this prognostic model were established through the use of receiver operating characteristic (ROC) curves.

Associations between immune cell infiltration and m6A-related lncRNAs were further assessed by utilizing the Tumor Immune Estimation Resource (TIMER) tool (https://cistrome.shinyapps.io/timer/), which assessed four types of immune cells (activated mast cells, memory B cells, T follicular helper cells, and resting memory CD4+ T cells). GISTIC 2.0 outcomes were implemented in the analyses of TIMER.

### 2.4. RNA Isolation and qRT-PCR

We collected twelve pairs of colorectal cancer tissues and adjacent normal tissues from patients who recently underwent surgical treatment in the Department of Gastrointestinal Surgery, Shanghai Changhai Hospital. Fresh tissues were frozen and stored at −80°C. This research was approved by the Medical Ethics Committee of Changhai Hospital of the Second Military Medical University. Informed consent was acquired from each involved patient. Total RNA from tissues of CRC patients was extracted using TRIzol reagent (Invitrogen, Carlsbad, CA, USA) according to the manufacturer's protocol. For complementary DNA (cDNA) synthesis, 1 *μ*g of total RNA and the PrimeScript RT reagent kit (Takara, Otsu, Shiga, Japan) were utilized. The SYBR Green assay (Takara) was used to perform qRT-PCR, and the progression was executed on a CFX-96 system (Bio-Rad Laboratories, Inc., Hercules, CA, USA). The GAPDH was used as an internal reference, and the relative lncRNA expression was calculated using the 2−∆∆Cq method. Primer sequences for qRT-PCR used in this study are shown in Supplementary Table S1.

### 2.5. Statistical Studies

GraphPad Prism 8.0, R v 3.60, and SPSS 24.0 (IBM, NY, USA) were employed for statistical assessments. Mann-Whitney *U* tests were employed to compare lncRNA expression in normal and tumor tissue samples. Data in different subgroups or groups were thoroughly evaluated via Student's *t*-test and one-way ANOVAs. Chi-square experiments were employed to assess categorical variables. The curves of Kaplan-Meier and log-rank assessments were utilized to compare survival outcomes. Pearson correlation analyses were used to explore the relationships among risk scores, PD-L1 status, levels of infiltration of immune cells, clinicopathological factors, and subtypes. The independent prognostic relevance of the scores of risk and other clinical traits was analyzed through the analyses of the multivariate and univariate Cox regression. ROC curves were implemented to appraise the predictive efficiency of m6A-related lncRNA signatures when estimating CRC patient OS. *p* < 0.05 was the significance threshold for the current research.

## 3. Results

### 3.1. m6A-Related lncRNA Identification in Patients with CRC

**Figure 1:**
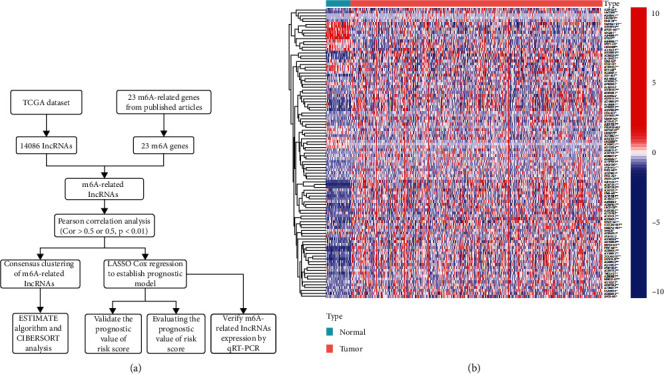
m6A-related lncRNA identification in patients with CRC. (a) Research flow chart. (b) Gene expression heatmap for 101 prognostic m6A-related lncRNAs in pairs of tumor and paracancerous tissues. ^∗^*p* < 0.05, ^∗∗^*p* < 0.01, and ^∗∗∗^*p* < 0.001.

We began by identifying and evaluating 14,086 lncRNAs present within the selected TCGA dataset. Initially, expression matrices for 23 m6A-associated genes were downloaded from the TCGA database, and those lncRNAs that correlated with the expression of one or more of these genes were defined as m6A-related lncRNAs (|Pearson R| > 0.5 and *p* < 0.001). Clustering analysis was conducted to separate cases in the TCGA-CRC cohort into different groups on the basis of their expression of m6A-related lncRNAs. The prognostic relevance of these m6A-related lncRNAs was then further evaluated through a series of univariate Cox regression analyses based upon a *p* < 0.05 cutoff within the analyzed TCGA datasets. A LASSO Cox analysis of the resultant 101 m6A-related prognostic lncRNAs was identified via this approach, with the overall workflow being detailed in Figure 1(a). Patterns of prognostic m6A-related lncRNA expression in CRC and normal tissues are shown in Figure 1(b).

### 3.2. m6A-Related lncRNA Identification and Evaluation of the CRC-Related Intratumoral Immune Cell Landscape

**Figure 2:**
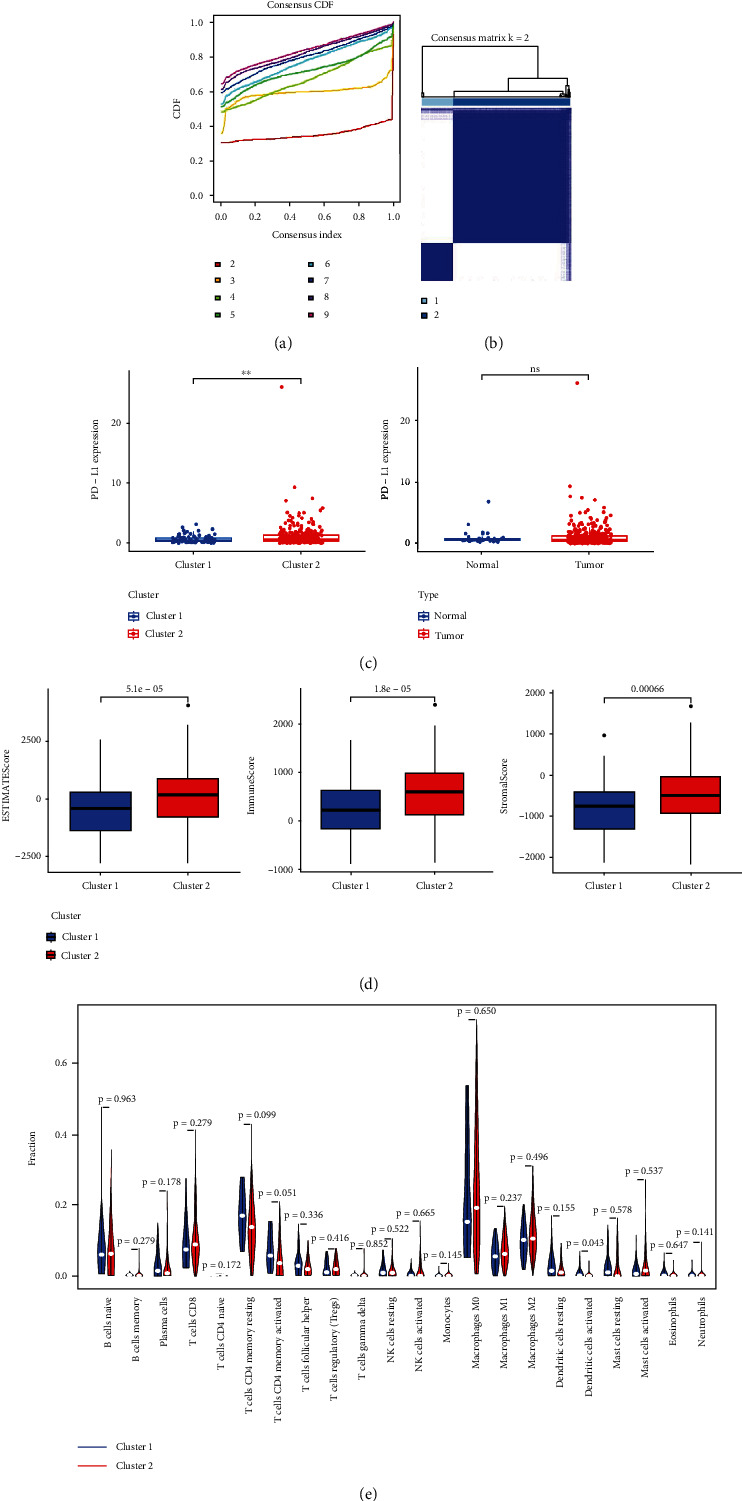
m6A-related lncRNA identification and evaluation of the CRC-related intratumoral immune cell landscape. (a) Consensus clustering matrix at *k* = 2. (b) Consensus clustering cumulative distribution functions (CDFs) and relative area under CDF curve from *k* values of 2 through 9. (c) PD-L1 expression in normal/tumor samples and in the cluster1/2 subtypes. (d) ESTIMATE, immune, and stromal scores in the cluster 1/2 subtypes. (e) Levels of predicted the infiltration of immune cells for 22 different immune cell subtypes in the cluster 1/2 subtypes. ^∗^*p* < 0.05 and ^∗∗^*p* < 0.01.

For this analysis, cumulative distribution functions (CDFs) were generated for consensus clusters for *k* values from 2-9 (Figure 2(a)), with the maximal area under the curve (AUC) value for this CDF function being evident at *k* = 2, at which time there was a clear difference in the expression of m6A-related lncRNAs between the two defined clusters (Figure 2(b)). The associations among m6A-related lncRNAs and PD-L1 expression were next assessed, revealing that the expression of PD-L1 was considerably greater in cluster 2 relative to cluster 1; there was also a trend toward increased PD-L1 expression in CRC tumor tissues relative to vicinal normal tissues (Figure 2(c)). Next, the algorithm of ESTIMATE was employed to measure stromal and immune scores for CRC case and tumor samples; these immune scores differed significantly between clusters, with higher immune ESTIMATE and stromal scores in cluster 2 patients relative to those in cluster 1 (Figure 2(d)). The CIBERSORT algorithm was utilized to evaluate the discrepancies in the levels of 22 populations of immune cells in these CRC tumors, revealing that there were high levels of M0 macrophages, CD8+ T cells, naïve B cells, and resting memory CD4+ T cells in cluster 2 patient tumors (Figure 2(e)).

### 3.3. Validation and Construction of a Prognostic m6A-Related lncRNA Risk Model

**Figure 3:**
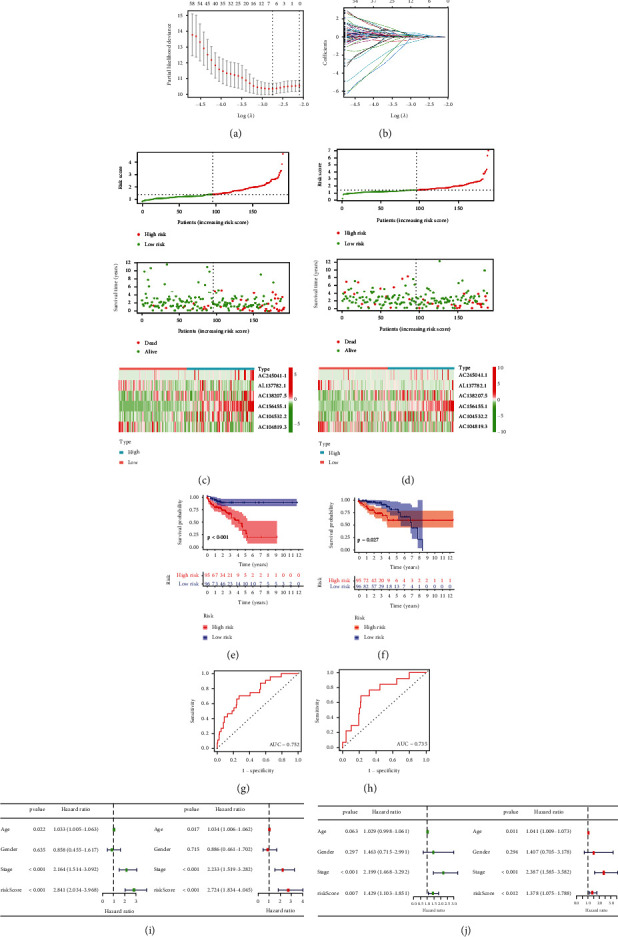
Validation and construction of a prognostic m6A-related lncRNA risk model. (a, b) Analysis of LASSO Cox regression for m6A-related lncRNAs. (c, d) Risk score, OS, and survival status distributions together with heatmaps demonstrating the expression of 6 prognostic m6A-Related lncRNAs in the training (c) and testing (d) TCGA cohorts. (e, f) CRC patient OS outcomes were compared based upon risk scores in the training (*p* < 0.001) and testing (*p* = 0.027) TCGA cohorts. (g, h) Risk score ROC curves for the TCGA training (g) and testing (h) cohorts. (i, j) Analyses of univariate and multivariate Cox regression for risk scores and other clinicopathological properties identifying independent prognostic indicators in the training (i) and testing (j) TCGA cohorts.

Risk scores were then measured based the regression coefficients derived from the LASSO algorithm for cases in both the TCGA testing and training cohorts (Figures 3(a) and 3(b)). Median risk score values were subsequently used to separate cases into high- and low-risk groups, and the patterns of OS and the expression of the six m6A-related lncRNAs composing the risk score were next assessed (Figures 3(c) and 3(d)). We found that low-risk CRC cases exhibited a longer OS relative to high-risk cases in either of the training and testing cohorts (Figures 3(e) and 3(f)). ROC curves further indicated that the developed m6A-LPS was able to reliably predict the OS of cases in both cohorts (Figures 3(g) and 3(h)). Univariate and multivariate analyses were then conducted, which confirmed that stage, age, and risk score values were all independent predictors of patient outcomes within the TCGA testing and training cohorts (Figures 3(i) and 3(j)).

### 3.4. Assessment of the Prognostic Utility of Risk Scores in Different CRC Patient Subgroups

**Figure 4:**
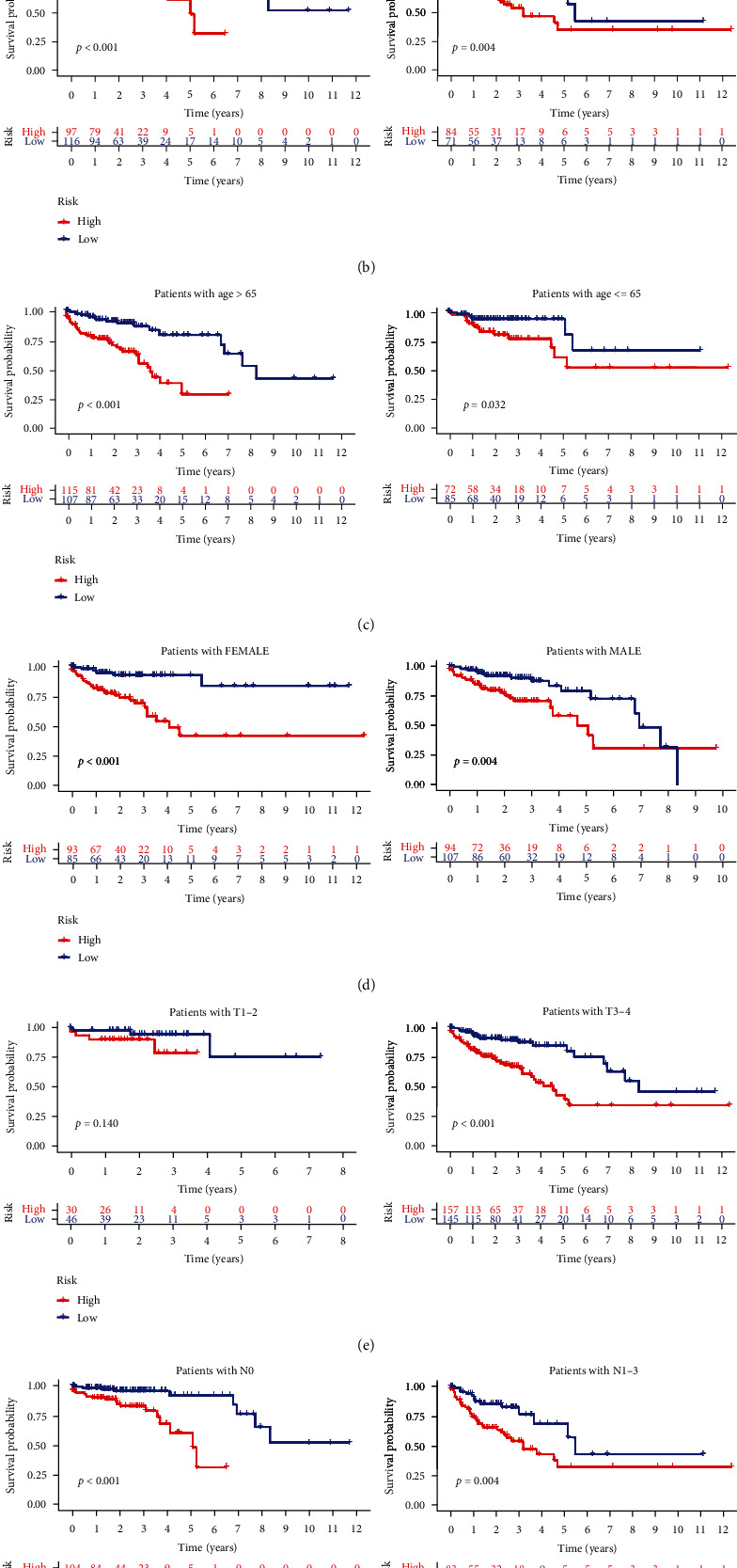
Assessment of the prognostic utility of risk scores in different CRC patient subgroups. (a) Low- and high-risk patient group clinicopathological findings and heatmaps. (b) Comparison of the survival of low- and high-risk cases in the dataset of TCGA (*cases* > 65 years old: *p* < 0.001 and *cases* ≤ 65 years old: *p* = 0.032). (c) Comparison of the survival of low- and high-risk cases in the dataset of TCGA (males: *p* = 0.004 and females: *p* < 0.001). (d) Comparison of the survival of low- and high-risk cases in the dataset of TCGA (cases with stage I-II disease: *p* < 0.001 and cases with stage III-IV disease: *p* = 0.004). (e) Comparison of the survival of low- and high-risk cases in the dataset of TCGA (cases with T1-2 disease: *p* = 0.140 and cases with T3-4 disease: *p* < 0.001). (f) Comparison of the survival of low- and high-risk cases in the dataset of TCGA (cases with N0 disease: *p* < 0.001 and cases with N1-3 disease: *p* = 0.004). (g) Comparison of the survival of low- and high-risk cases in the dataset of TCGA (M0 cases: *p* < 0.001 and cases with M1: *p* = 0.015). All survival outcomes were compared through curves of Kaplan-Meier and log-rank experiments.

Next, we examined the relationship between risk scores and CRC patient clinical features. Heatmaps were used to evaluate the patterns of expression of the six m6A-related lncRNAs in low- and high-risk cases (Figure 4(a)). This revealed that AL137782.1 and AC104819.3 were expressed at lower levels within the high-risk group relative to the low-risk group, whereas AC245041.1, AC138207.5, AC156455.1, and AC104532.2 exhibited the opposite trend. To more fully understand the prognostic values of these risk scores, we stratified CRC cases based upon their disease status and found that compared to low-risk cases, those in the high-risk group exhibited worse OS for both individuals with stage I-II and stage III-IV disease (Figure 4(b)). Similarly, these prognostic m6A-related lncRNAs were also able to estimate the OS of cases irrespective of age (>65 vs. ≤65 years) (Figure 4(c)), gender (female vs. male) (Figure 4(d)), T status (T1-2 vs. T3-4) (Figure 4(e)), N status (N0 vs. N1-3) (Figure 4(f)), and M status (M0 vs. M1) (Figure 4(g)).

### 3.5. Association between Risk Scores and Immune Cell Infiltration

**Figure 5:**
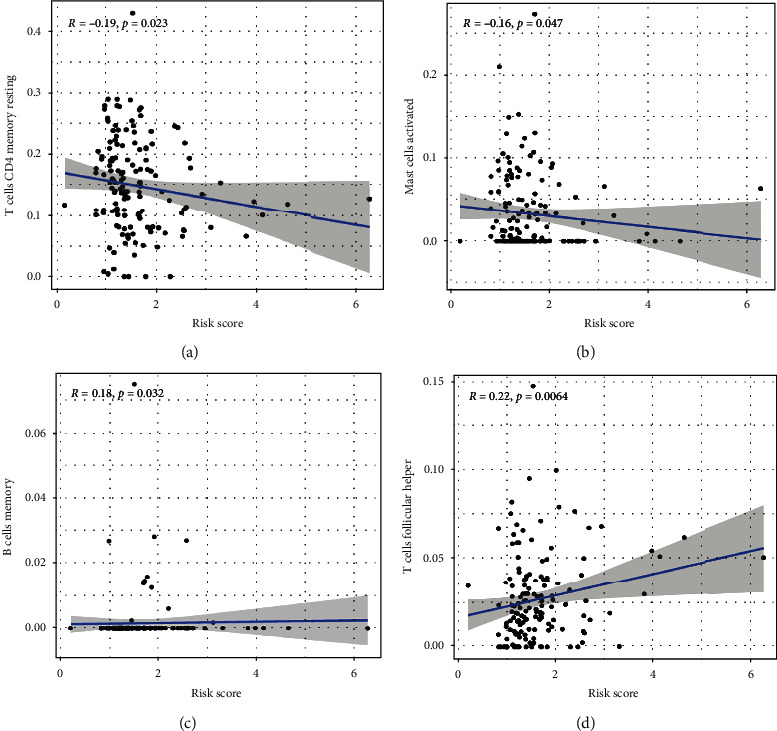
Associations between risk scores and immune cell infiltration. (a–d) Memory B cells (a), activated mast cells (b), resting CD4 memory T cells (c), and T follicular helper cells (d).

We explored the associations between risk scores and intratumoral infiltration by four different immune cell types to meticulously discover the influence of the 6 m6A-related lncRNAs composing our risk signature and the TIME in CRC. Risk scores are considerably negatively related to infiltration by resting memory CD4+ T cells (*p* = 0.023) and activated mast cells (Figures 5(a) and 5(b)), whereas they are positively correlated with infiltration by memory B cells (*p* = 0.032) and T follicular helper cells (Figures 5(c) and 5(d)).

### 3.6. Validation of the Expression Levels of lncRNAs AC156455.1 and AC104532.2 with Prognostic Signature

**Figure 6:**
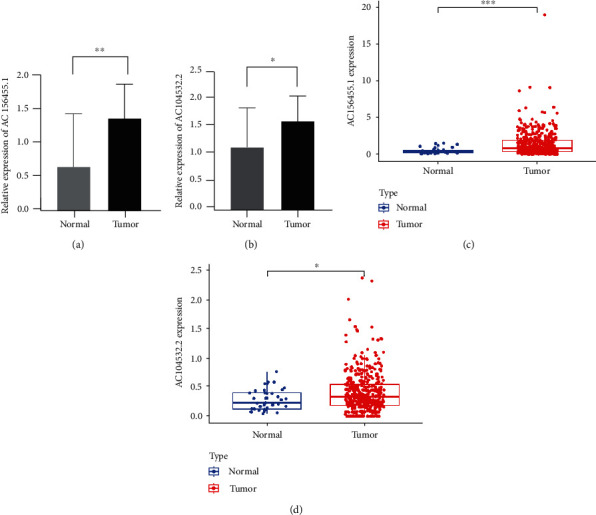
Validation of the expression Levels of lncRNAs AC156455.1 and AC104532.2 of prognostic signature. (a, b) AC156455.1 and AC104532.2 expressions in twelve colorectal cancer tissues and twelve adjacent normal tissues using RT-qPCR. (c, d) AC156455.1 and AC104532.2 expressions in colorectal cancer tissues compared with corresponding normal tissues from TCGA database. ^∗^*p* < 0.05 and ^∗∗^*p* < 0.01.

To further verify the accuracy of the m6A-related lncRNA signature, the expression levels of lncRNAs AC156455.1 and AC104532.2 were measured in twelve colorectal cancer tissues and twelve adjacent normal tissues using qRT-PCR. lncRNAs AC156455.1 and AC104532.2 were upregulated in colorectal cancer tissues compared with corresponding normal tissues (Figures 6(a) and 6(b)). Meanwhile, the same results were analyzed; lncRNAs AC156455.1 and AC104532.2 were also upregulated in colorectal cancer tissues compared with corresponding normal tissues from TCGA database (Figures 6(c) and 6(d)).

## 4. Discussion

Several prior reports have explored the link between m6A modifications and the regulation of cancer pathogenesis [24–28], but the mechanisms whereby lncRNAs may shape this relationship are yet to be defined. KIAA1429 has been indicated to drive the progression of liver cancer through the m6A modification of the lncRNA GATA3 [29]. In glioblastoma stem cells, the lncRNA FOXM1-AS has been illustrated to influence interactions among FOXM1 and ALKBH5 to shape cell maintenance [24]. In light of the above results, we speculate that m6A modifications of specific mRNAs may shape oncogenesis, and as such, further study of the impact of such m6A modifications on lncRNA function is warranted to better identify key therapeutic targets or prognostic biomarkers associated with particular cancers. LINC00265 has been shown to predict undesirable findings in cases with AML [30], while LINC00665 has been associated with enhanced activation of the pathway of PKR/NF-*κ*B hepatocellular carcinoma and with concomitant increases in malignancy [31], while in gastric cancer, this same lncRNA can activate the Wnt pathway to promote tumor progression [32]. In our study, we explored the prognostic relevance of m6A-related lncRNAs by analyzing data from 437 CRC patients in the TCGA database. We ultimately defined 101 prognostically relevant m6A-related lncRNAs, of which 6 were employed to establish an m6A-related lncRNA prognostic signature (m6A-LPS) capable of estimating the OS of patients with CRC. When stratified into low- and high-risk groups following the median risk score values, high-risk CRC patients survived for significantly shorter periods relative to low-risk patients. Multivariate analyses further indicated that these m6A-LPS values were independent predictors of CRC patient OS. While several of the lncRNAs within our risk signature have been studied in oncogenic contexts, they have not been analyzed in the context of CRC, and there have been few reports regarding interactions between these lncRNAs and m6A-associated genes. As such, our findings offer novel insights regarding lncRNAs targeted by m6A regulators in the context of CRC, potentially shedding new light on their ability to promote CRC onset and progression.

The heterogeneous tumor microenvironment (TME) often harbors a diverse array of immunosuppressive signals, shaping tumor development, patient prognosis, and therapeutic responses [33–36]. The TME consists of an assorted immune cells, vascular structures, and stromal cells, all of which can impact the oncogenic progression associated with a given tumor type. Immune cell infiltration within the TME can predict patient outcomes and is often correlated with tumor grade, stage, and metastasis [37, 38]. For example, tumor-associated macrophages (TAMs) can generate immunosuppressive cytokines including TGF-B and IL-10 for example, which can drive preferential tumor outgrowth and contribute to poor patient outcomes [39–41]. More potent tumor infiltration by CD4+ and CD8+ T cells, on the contrary, is often related to better patient survival and a higher response rate to immunotherapy [42]. We observed that PD-L1 expression levels in cluster 2 were considerably greater relative to cluster 1, and a trend towards increased PD-L1 expression in CRC tissues relative to normal tissues. It is critical that consensus criteria be established in order to determine the CRC cases that are most probable to respond to immunotherapeutic treatment. We found that the ESTIMATE, stromal, and immune scores of cluster 2 were greater than those in cluster 1. This strongly suggests a close relationship between patterns of m6A-related lncRNA expression and the ability of particular immune cells to enter or persist within the TIME, thereby altering patient responses to immunotherapeutic intervention. Risk scores were all negatively associated with activated mast cell and resting memory CD4+ T cell infiltration in this study, whereas they were positively associated with memory B cell and T follicular helper cell infiltration. We reported that lncRNAs AC156455.1 and AC104532.2 were upregulated in colorectal cancer tissues compared with corresponding normal tissues using qRT-PCR, compared with previous studies. Therefore, lncRNAs AC156455.1 and AC104532.2 can be used as biomarkers for diagnostic and prognosis in CRC, to provide new targets for future immunotherapy.

## 5. Conclusions

In summary, we herein conducted a systematic assessment of the prognostic relevance of m6A-associated lncRNAs in CRC and explored their associations with PD-L1 expression and the TIME. Risk scores derived from m6A-associated lncRNA-based expression signatures were also found to be independently related to CRC patient prognosis, and further predictive analyses suggested that these lncRNAs may be associated with the regulation of the TIME in CRC tumors. As such, lncRNAs AC156455.1 and AC104532.2 associated with tumor immune responses have the potential to guide the design of modern immunotherapeutic treatments for CRC.

## Data Availability

All data generated or analyzed during this study are included in this published article.

## Abbreviations

M6A: N6-methyladenosine
TIME: Tumor immune microenvironment
TCGA: The Cancer Genome Atlas
CRC: Colorectal cancer
LASSO: Least absolute shrinkage and selection operator
M6A-LPS: m6A-related lncRNA prognostic signature
siRNA: Small interfering RNAs
lncRNAs: Long noncoding RNAs
PD-L1: Programmed death-ligand 1
ROC: Receiver operating characteristics
FDR: False discovery rate
TIMER: Tumor Immune Estimation Resource
CDFs: Cumulative distribution functions
AUC: Area under the curve
GSEA: Gene set enrichment analysis
OS: Overall survival
TAMs: Tumor-associated macrophages.

## Supplementary Materials

Supplementary Materials Table S1: primer sequences for qRT-PCR used in this study.
