# Supplementary material for: lncRNAs AC156455.1 and AC104532.2 as Biomarkers for Diagnosis and Prognosis in Colorectal Cancer
Source: Dis Markers. 2022 Oct 13;2022:4872001. doi: 10.1155/2022/4872001 (PMC9584711; doi:10.1155/2022/4872001)
Supplement: Supplementary Materials — Table S1: primer sequences for qRT-PCR used in this study. [file 4872001.f1.docx]

Table S1

Primer sequences for qRT-PCR

| GAPDH | FP:  CTGGGCTACACTGAGCACC | RP: AAGTGGTCGTTGAGGGCAATG |
| --- | --- | --- |
| AC156455.1 | FP: CCCAGTCCTATCCAATCACC | RP:  TTCTTTCCCACCAGTTCCC |
| AC104532.2 | FP:  ACAGCAACAAAAATGCCCC | RP:  TTCAAGCGATTCTCCTGCC |
